# Intra-articular injection of expanded autologous bone marrow mesenchymal cells in moderate and severe knee osteoarthritis is safe: a phase I/II study

**DOI:** 10.1186/s13018-017-0689-6

**Published:** 2017-12-12

**Authors:** Mahasen Al-Najar, Hiba Khalil, Jihad Al-Ajlouni, Eman Al-Antary, Mohammad Hamdan, Reem Rahmeh, Dana Alhattab, Osama Samara, Mohamad Yasin, Amenah Al Abdullah, Esraa Al-jabbari, Dima Hmaid, Hanan Jafar, Abdalla Awidi

**Affiliations:** 1 Department of Radiology, School of Medicine, University of Jordan, Amman, Jordan; 2 Department of Hematology, Faculty of Medical Laboratory Sciences, Alneelain University, Khartoum, Sudan; 3 Department of Orthopedics, School of Medicine, University of Jordan, Amman, Jordan; 4 Cell Therapy Center, University of Jordan, Amman, Jordan; 5 School of Medicine, University of Jordan, Amman, Jordan; 6 Department of Medicine, School of Medicine, Amman, Jordan

**Keywords:** Knee osteoarthritis, Mesenchymal stem cells, Bone marrow, KOOS score, Intra-articular injection

## Abstract

**Background:**

Knee osteoarthritis (KOA) is a major health problem especially in the aging population. There is a need for safe treatment that restores the cartilage and reduces the symptoms. The use of stem cells is emerging as a possible option for the moderate and severe cases. This study aimed at testing the safety of autologous bone marrow mesenchymal stem cells (BM-MSCs) expanded in vitro when given intra-articularly to patients with stage II and III KOA. As a secondary end point, the study tested the ability of these cells to relieve symptoms and restore the knee cartilage in these patients as judged by normalized knee injury and Osteoarthritis Outcome Score (KOOS) and by magnetic resonance imaging (MRI).

**Methods:**

Thirteen patients with a mean age of 50 years suffering from KOA stages II and III were given two doses of BM-MSCs 1 month apart totaling 61 × 10^6^ ± 0.6 × 10^6^ by intra-articular injection in a phase I prospective clinical trial. Each patient was followed for a minimum of 24 months for any adverse events and for clinical outcome using normalized KOOS. Cartilage thickness was assessed by quantitative MRI T2 at 12 months of follow-up.

**Results:**

No severe adverse events were reported up to 24 months follow-up. Normalized KOOS improved significantly. Mean knee cartilage thickness measured by MRI improved significantly.

**Conclusion:**

BM-MSCs given intra-articularly are safe in knee osteoarthrosis. Despite the limited number of patients in this study, the procedure described significantly improved the KOOS and knee cartilage thickness, indicating that they may enhance the functional outcome as well as the structural component.

**Trial registration:**

ClinicalTrials.gov, NCT02118519

## Background

Knee osteoarthritis (KOA) is a common condition affecting the adult population causing pain and dysfunction of the knee joint. Subsequently, there is a negative impact on the quality of life of these patients [1, 2].

It is estimated that 9–14 million adults in the USA suffer from symptomatic or radiographic knee osteoarthritis, especially in subjects above the age of 65 years. However, the incidence is increasing among population younger than 65 [1, 3, 4]. Many studies showed KOA to be common among the adult population worldwide especially individuals older than 65 causing significant disease burden [5]. Pharmacological approach to the treatment of KOA is well established in most guidelines and has been extensively outlined [6]. Cellular therapy is an emerging modality for the treatment of KOA.

A recent meta-analysis of the 11 trials with 558 patients using mesenchymal stem cells (MSCs) was published [7]. There was an improvement in various clinical scores. The authors concluded that there was no significant difference in the comprehensive evaluation index after stem cell treatment, despite the significant improvement in clinical symptoms and cartilage morphology [8]. A recent phase I-II of expanded autologous bone marrow stem cells has been published [9]. It reported the safety and effectiveness of this modality. There is one published work using allogeneic bone marrow-derived MSCs in advanced KOA in humans showing clinical improvement but no significant MRI improvement [10].

In this paper, we report on the results of 13 patients who were treated by expanded autologous bone marrow mesenchymal stem cells (BM-MSCs) in an open-label prospective study and followed for 2 years for any adverse events and for efficacy by normalized Knee Osteoarthritis Outcome Score (KOOS) and by MRI.

## Methods

### Patients

This is a phase I prospective open-label safety study. The study was prospectively registered in clinicaltrial.gov (reference NCT02118519).

After an IRB approval, a signed informed consent was obtained in accordance with the latest version of Helsinki Declaration. From February 2014 to August 2014, 13 adult patients, 7 females and 6 males with moderate to moderately severe knee, were enrolled in a prospective study using autologous expanded bone marrow mesenchymal stem cells (BM-MSCs) delivered by percutaneous intra-articular injection using lateral tibio-femoral approach by an experienced orthopedic surgeon.

KOA staging was done in accordance with the Kellgren and Lawrence classification [11] using standard knee x-ray imaging with the standing anteroposterior projection and horizontal lateral projection. Image interpretation and staging were independently done by two radiologists. No patient with significant varus or valgus malalignment or significant effusion of either knee or both knees was included.

Inclusion and exclusion criteria are shown in Table 1.

**Table 1:** Inclusion and exclusion criteria

Inclusion criteria	Exclusion criteria
1. Chronic knee joint pain and or swelling (more than 6 months) 2. Grade II–III KOA confirmed by two observers 3. Absence of local or systemic infection 4. Absence of significant hematological disease 5. Absence of significant biochemical or hematological laboratory tests abnormalities 6. Informed consent form signed by the patient	1. Age less than 18 or older than 65 years 2. Intra-articular treatment in the past 6 months 3. Significant deformity of the knee 4. Knee ligament injury or ruptured meniscus observed by MRI 5. Infection or positive serology for transmissible agents 6. Body mass index (BMI) greater than 30.5 7. Women in childbearing age 8. Malignancy 9. Immunosuppressive drugs

Bone marrow (BM) aspirate was done in an outpatient setting using local anesthetic of 2% lidocaine. A total of 35–40 ml of bone marrow was obtained in multiple small aspirate of 3–5 ml each from the iliac crest. The samples were collected in sterile citrated tubes of 3.8%. Prior to collection, the patient had to have normal prothrombin time (PT), partial thromboplastin time (PTT), and platelet count.

### BM-MSC isolation and culture

BM aspirates were diluted in a 1:1 ratio with phosphate-buffered saline (PBS) pH 7.4 (Gibco, USA, Cat # 10010-015). Mononuclear cells (MNCs) were separated by density gradient centrifugation using Ficoll-Paque (Histopaque 1077, Sigma, Cat. 10771). MNCs were counted and seeded at a density of 0.16 × 10^6^ cells/cm^2^ in T1 75 cm^2^ tissue culture flask (NUNC, USA) in complete media. The complete media consist of α-minimum essential medium (α-MEM) (Gibco, Cat. 22561-021) supplemented with 100 IU penicillin and 100 IU streptomycin (Gibco), 10% FBS qualified (Gibco, Cat. 12763017), and 2 Mm L-glutamine (Gibco, Cat. 25030081). Cells were allowed to attach for 24 h before changing media. Subsequently, the culture medium was changed twice a week. When cultures reached 70–80% confluence, subculturing was performed using trypsin-EDTA 0.25% (Gibco, USA, Cat. 25200056). After the primary passage, cells were seeded at a density of 4 × 10^3^ cells/cm^2^. Cells were cultured until the number reaches the clinical grade with an average number of 30.5 × 10^6^ cells per dose injected per patient. At harvest, cells of all patients were in passages lower or equal to 4. Prior to injection, MSCs were tested for endotoxin, mycoplasma, and microbial contamination. For injection, MSCs were washed and suspended in 5 ml 0.9% normal saline.

### Characterization of BM-MSCs

#### Flow cytometry analysis

Surface marker characterization for MSCs isolated from all patients was performed in accordance with the International Society for Cellular Therapy (ISCT) recommendations [12]. BD Stemflow™ hMSC Analysis Kit (BD, USA) was used according to the manufacturer instructions. Passage 3 cells were stained with antibodies against CD73, CD90, CD105, CD44, CD34, CD11b, CD19, CD45, and HLA-DR. Corresponding mouse isotype antibodies were used as a control. Canto BD II flow cytometer instrument (BD, USA) was used for running samples. Diva software (BD, USA) was used for data analyses. The percentage of expressed cell surface markers was calculated from 10,000 gated cells.

#### Differentiation potential

Assessment of adipogenic and osteogenic differentiation potential for MSCs isolated from randomly selected patient samples were performed in accordance with the ISCT recommendation. StemPro® Adipogenesis and Osteogenesis Differentiation Kit (Gibco, USA) was used according to the manufacturer instructions. Cells at passages 3–5 were used in differentiation experiments. To detect adipogenic differentiation, oil red O stain was used. To detect osteogenic differentiation, alizarin red S stain was used.

#### Percutaneous intra-articular delivery of BM-MSCs

The expanded BM-MSCs were washed and suspended in 5 ml 0.9% normal saline. The skin was prepared by aseptic technique with 1% chlorhexidine in alcohol or iodine solution. The cells were delivered percutaneously into the knee joint using lateral tibio-femoral approach by an experienced orthopedic surgeon. Total of two injections were given 1 month apart. Patients were asked to stop all analgesic medication and only allowed paracetamol as needed to alleviate the pain if any.

### Clinical assessment

Patients were followed for adverse events by direct questioning on days 1, 7, 14, 28, 60, and then every 6 months until month 24. The patient was assessed by physical examination just before the second injection and 2 months after the second injection. Blood count and clinical chemistry were done 3 and 24 months after the first injection. For outcome, normalized KOOS [13] was used at baseline before injections and at months 1, 2, 4, 6, 12, and 24 after the first injection.

### MRI

MRI scans were done at baseline, 6 and 12 months using 3 T Siemens scanner. Standard knee MRI imaging protocol was obtained in axial, coronal, and sagittal planes, in addition to using a specific cartilage sequence which is T1-weighted FS spoiled 3D gradient echo in axial and sagittal planes. Detailed measurements were obtained from each compartment from three points: anterior, central, and posterior. The mean thickness was calculated. Identical sequences and measurement sites were done on the follow-up scans.

### Statistical analysis

For statistical analysis, IBM SPSS software version 20 was used. The data was described as mean and standard deviation. Univariate analysis between baseline and pre-specified time points was performed. Confidence interval was set at 95%.

## Results

### Characterization of BM-MSCs

**Fig. 1:**
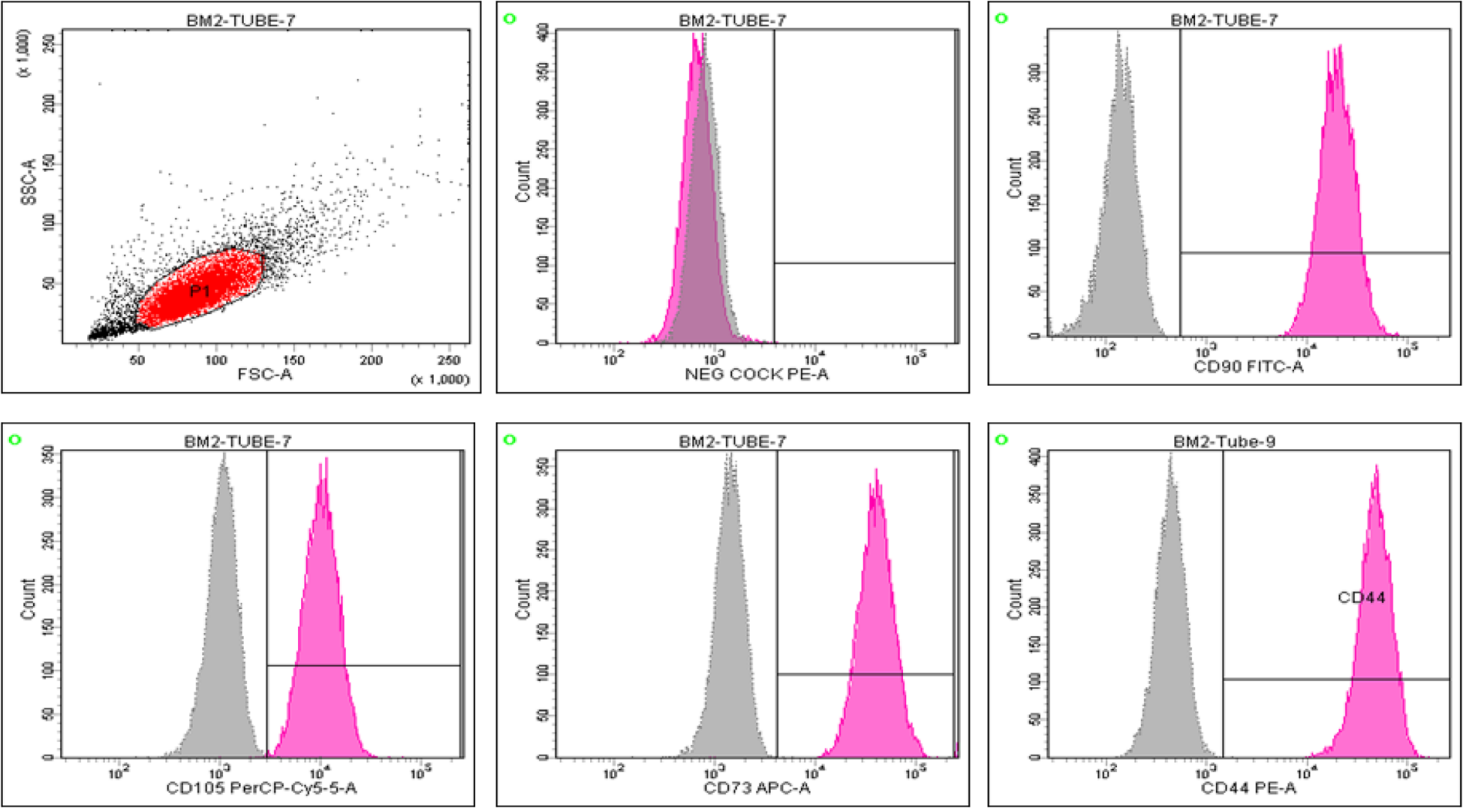
Flow cytometry analysis of BM-MSCs surface marker expression

All isolated patients of BM-MSCs were positive for MSC signature markers determined by ISCT; CD90, CD105, CD73, and CD44 and were negative for CD34, CD45, CD11b, CD19, and HLA-DR (Fig. 1).

The data shown are the representative cell phenotype analyzed at passage 4. Gray peaks correspond to the isotype control, and the pink peaks to the antibody of interest.

**Fig. 2:**
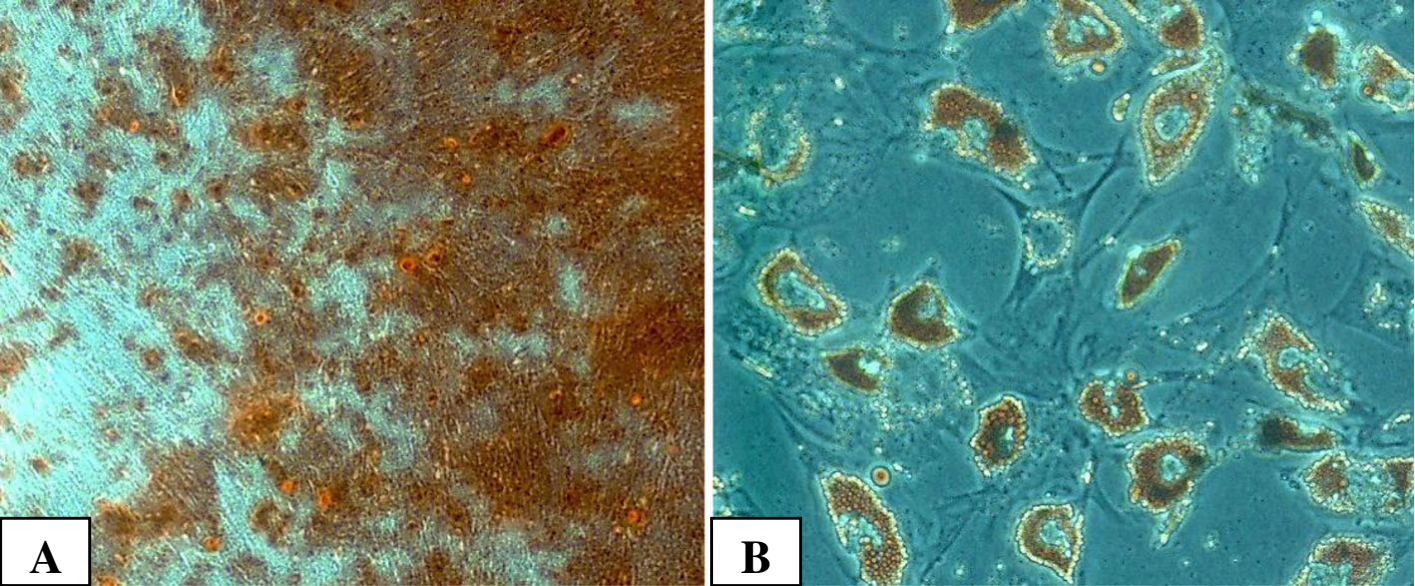
Representative sample of BM-MSCs. **A** Osteogenic and **B** adipogenic differentiation

Upon induction of differentiation, BM-MSCs were differentiated into adipocytes and osteocytes (Fig. 2).

### Patients

Total of 13 adult patients were enrolled, 7 females and 6 males, with stage II (5 patients) and stage III (8 patients) with mean age of 50 years (range 34–63 years were included in the study. Details of patients are shown in Table 2.

**Table 2:** Patients’ characteristics and dose of MSC injected

Variables	Mean (range)
Age/years	50 (34–63)
Gender (F/M)	7/6
KOA stage (II/III)	5/8
Cell dose in 1st treatment (10^6^)	30.8 (28–35)
Cell dose in 2nd treatment (10^6^)	30.4 (26–33)
Time interval/days between treatments	92.3 (90–110)

### Safety outcome

There were total of three adverse events; all were local events. Two instances in which patients had pain in the injected joint within 2 h which needed cold compresses and resting the joint for several hours. The pain disappeared within 24 h. There was one instance in which the patient developed moderate pain and mild knee swelling 6 h after receiving the injection needing cold compresses and mild oral analgesia. The pain disappeared within 48 h. No clinical or biochemical adverse events were noticed after 2 years of follow-up.

### Outcome as measured by normalized KOOS

Table 3 shows the positive changes related to all five major areas measured by normalized KOOS. All were significantly better at 6, 12, and 24 months post first injection.

**Table 3:** Univariate analysis of normalized KOOS for 13 patients suffering from KOA treated with BM-MSCs

Normalized KOOS sections	Baseline (mean)	6th month follow-up (mean)	*P* value	1 year follow-up (mean)	*P* value	2 years follow-up (mean)	*P* value
Symptoms	67.300	91.2308	0.000	89.9	0.000	88.7	0.000
Pain	62.585	89.0538	0.000	89.7	0.000	89.4	0.000
Daily life activity	64.223	90.8308	0.000	92.2	0.000	93	0.000
Sport	40.25	79.9769	0.000	81.1	0.000	81.6	0.000
Quality of life	34.162	75.4923	0.000	76.9	0.000	77.4	0.000

### Results of knee cartilage thickness as measured by MRI

At 6 months, there was no significant change in the cartilage thickness by MRI. At 12 months, a significant improvement in the thickness of knee cartilage in the femoral and tibia plates was noticed as shown in Table 4. Only one female patient deteriorated by MRI despite of KOOS improvement.

**Table 4:** Changes in knee cartilage thickness after 12 months of receiving the 1st injection

Variable	Baseline (T1)	SD	SE	After 12 months of treatment (T2)	SD	SE	*P* value (two-tailed)
Mean tibial plate thickness in mm	*2.15*	*0.67*	*0.076*	*2.38*	*0.63*	*0.072*	*.000*
Mean femoral plate thickness in mm	2.16	0.78	0.09	2.5	0.76	0.086	.000

Mean baseline in mm = 2.15; mean at 12 months in mm = 2.45

## Discussion

Knee cartilage has limited regenerative capacity [14]. Mesenchymal stem cells (MSCs) are known to have paracrine and differentiation properties. They can produce extracellular matrix within the joint. These properties make them good target for use in the regeneration of knee cartilage [15–19].

Whether MSCs stimulate the proliferation and differentiation of resident progenitor cells or they differentiate into chondrocytes remains to be clarified [15]. Rabbit and goat models of osteoarthrosis suggest that the repair occurs through paracrine effects by stimulation of endogenous repair mechanisms [20]. There is a great need to explore new methods to treat KOA which are safer than current pharmacological approaches. The pharmacological therapy has numerous limitations, including serious gastrointestinal, renal, and cardiac adverse events; some of which are life threatening or may leave a permanent disability [21–23].

Several studies have been or are being conducted to find alternatives to pharmacological therapy, including platelet-rich plasma, platelet lysate, and mesenchymal stem cells of either bone marrow or adipose origin [24–29]. Increasingly, these studies are showing the safety of MSCs in KOA. A common aim for researchers in this field is to restore the knee cartilage via non-invasive procedures such as cell and/or tissue transplantation [30]. Additionally, the source and dose of MSCs are still to be established [27, 28]. A further point of concern is that most studies report short-term safety, rather than long-term follow-up.

This work shows that the use of BM-MSCs is safe with only minimal early pain in some patients in the injected joint which resolved quickly without any intermediate or long-term clinical or biochemical adverse events. Bone marrow is attractive since it can easily be harvested as an outpatient procedure and without the need for patient hospitalization. Patients were followed up for 2 years.

The work also provides preliminary evidence that BM-MSCs are effective in KOA, as judged by the significant improvement in KOOS and by MRI. All symptoms significantly improved conferring significant improvement in the quality of life of these patients with grade II and III KOA. However, we wish to emphasize that the small number of participants in this study prohibits generalization of efficacy, and further work is warranted.

Although the number of the patients is small, they add to our current evidence gathering of safety. Of note, our patients were followed for 24 months. A placebo-controlled trial with sufficient number of patients is needed to establish the long-term efficacy and disease-modifying properties of BM-MSC.

We suggest that next trials should also explore the dose of MSC and the source of MSC. There is a need to establish the safety of allogeneic MSC for KOA. The use of allogenic MSC can be standardized and the dose can be better controlled, and the cell variability can be reduced to the minimum. We believe that MSCs are potential definitive therapy for KOA.

## Conclusion

This work showed that in vitro expanded autologous bone marrow-derived mesenchymal stem cells are safe and tolerable when injected intra-articularly for knee osteoarthritis patients. Preliminary data of efficacy were also presented, as measured by both the KOOS score as well as MRI changes. Safety and efficacy were established for more than 2 years of follow-up. Further work is needed to provide sufficient evidence of efficacy.

## Abbreviations

BM: Bone marrow
BM-MSCs: Bone marrow mesenchymal stem cells
IRB: Institutional review board
ISCT: International Society for Cellular Therapy
KOA: Knee osteoarthritis
KOOS: Knee Osteoarthritis Outcome Score
MNCs: Mononuclear cells
MRI: Magnetic resonance imaging
MSCs: Mesenchymal stem cells
PBS: Phosphate-buffered saline
PT: Prothrombin time
PTT: Partial thromboplastin time
α-MEM: α-Minimum essential medium
